# Limited Polymorphism of the Kelch Propeller Domain in Plasmodium malariae and P. ovale Isolates from Thailand

**DOI:** 10.1128/AAC.00138-16

**Published:** 2016-06-20

**Authors:** Supatchara Nakeesathit, Naowarat Saralamba, Sasithon Pukrittayakamee, Arjen Dondorp, Francois Nosten, Nicholas J. White, Mallika Imwong

**Affiliations:** a Mahidol Oxford Research Unit, Faculty of Tropical Medicine, Mahidol University, Bangkok, Thailand; b Department of Clinical Tropical Medicine, Faculty of Tropical Medicine, Mahidol University, Bangkok, Thailand; c Department of Molecular Tropical Medicine and Genetics, Faculty of Tropical Medicine, Mahidol University, Bangkok, Thailand; d Centre for Tropical Medicine, Nuffield Department of Medicine, Churchill Hospital, Oxford, United Kingdom; e Shoklo Malaria Research Unit, Tak, Thailand

## Abstract

Artemisinin resistance in Plasmodium falciparum, the agent of severe malaria, is currently a major obstacle to malaria control in Southeast Asia. A gene named “*kelch13*” has been associated with artemisinin resistance in P. falciparum. The orthologue of the *kelch* gene in P. vivax was identified and a small number of mutations were found in previous studies. The *kelch* orthologues in the other two human malaria parasites, P. malariae and P. ovale, have not yet been studied. Therefore, in this study, the orthologous *kelch* genes of P. malariae, P. ovale wallikeri, and P. ovale curtisi were isolated and analyzed for the first time. The homologies of the *kelch* genes of P. malariae and P. ovale were 84.8% and 82.7%, respectively, compared to the gene in P. falciparum. *kelch* polymorphisms were studied in 13 *P. malariae* and 5 P. ovale isolates from Thailand. There were 2 nonsynonymous mutations found in these samples. One mutation was P533L, which was found in 1 of 13 P. malariae isolates, and the other was K137R, found in 1 isolate of P. ovale wallikeri (*n* = 4). This result needs to be considered in the context of widespread artemisinin used within the region; their functional consequences for artemisinin sensitivity in P. malariae and P. ovale will need to be elucidated.

## INTRODUCTION

Malaria remains one of the world's most important infectious diseases, with an estimated 198 million cases and 584,000 deaths in 2013 (1). Malaria is caused by protozoa of the genus Plasmodium, with the following five species causing disease in humans: Plasmodium falciparum, P. vivax, P. malariae, P. ovale, and P. knowlesi. P. malariae is the third most common infecting species, with incidences in areas of endemicity reported to be <4% to 20% of the total number of malaria infections (2). This parasite has a 72-h erythrocytic developmental cycle and is usually detected at low parasitemias in mixed infections with either P. falciparum or P. vivax. Although P. malariae does not form liver hypnozoites, it can persist in the circulation for many years. Infections with P. ovale are found in sub-Saharan Africa, the Middle East, Papua New Guinea, and Southeast Asia. P. ovale is a less common parasite, but it still has an estimated global incidence in sub-Saharan Africa exceeding 15 million cases annually (3). Like P. malariae, P. ovale causes infections with low parasitemias and is usually found with P. falciparum or P. vivax. P. ovale can cause relapse infections from dormant exoerythrocytic-stage parasites in the liver, called hypnozoites (4).

**FIG 1:**
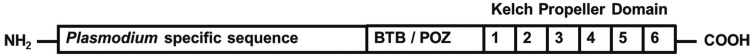
Structure of the Plasmodium sp. *kelch* gene.

Artemisinin-based combination therapies (ACTs) have been adopted as the first line of treatment for uncomplicated falciparum malaria in most countries where malaria is endemic, including Thailand (1). However, over the last few years, artemisinin-resistant P. falciparum has emerged in western Cambodia and is now firmly established in the surrounding countries, including Thailand, Laos, Vietnam, and Myanmar. Recently, a molecular marker for artemisinin resistance was identified, namely, mutations in the P. falciparum kelch (*Pfkelch*) gene, which is located on chromosome 13 and encodes a 727-amino-acid protein. Mutations in the so-called propeller region of the Kelch protein (K13 propeller) closely correlate with delayed parasite clearance, which defines the artemisinin resistance phenotype. The *kelch* gene consists of three domains: a Plasmodium-specific domain, a BTB/POZ domain, and the *kelch* propeller domain (Fig. 1). The original studies reported the K13 propeller mutations C580Y, R539T, and Y493H in Cambodian P. falciparum strains, and interestingly, only one mutation per *kelch* gene seemed to be allowed (5). Since these initial studies, more than 60 single nucleotide polymorphisms (SNPs) in the K13 propeller have been identified, of which most correlate with the slow-clearance phenotype (6). Among Cambodian P. vivax strains, a V552I polymorphism in the orthologous *kelch* gene has been described for two isolates (7). It is not known whether this mutation in P. vivax causes resistance to artemisinins. Because P. malariae and P. ovale are frequently found as mixed infections with P. falciparum, these species are also exposed to ACTs when P. falciparum infections are being treated. It is therefore plausible that *kelch* mutations in P. malariae and P. ovale might have emerged and may confer artemisinin resistance. The *kelch* genes from these two parasites have not been isolated or studied previously. The present study aimed to isolate the “propeller region” of the *Pmkelch* and *Pokelch* genes, from P. malariae and P. ovale, respectively, and to determine the presence of polymorphisms in these genes.

## MATERIALS AND METHODS

P. malariae and P. ovale strains were obtained from blood samples taken from previous studies performed in Thailand between 1995 and 2012 (*n* = 18), under ethical approval MUTM2011-049-05. This study was reviewed and approved by the Ethics Committee of the Faculty of Tropical Medicine, Mahidol University, Thailand, with ethical approval MUTM2015-001-01. All 18 P. malariae and P. ovale isolates included in this study were from monoinfections with parasitemias varying from 26/500 white blood cells (WBC) to 1/1,000 red blood cells (RBC). Patients were treated with chloroquine according to standard guidelines. DNA extraction was performed with a DNA minikit (Qiagen, Germany) following the manufacturer's instructions. Genomic DNAs were kept at −20°C until further use.

**FIG 2:**
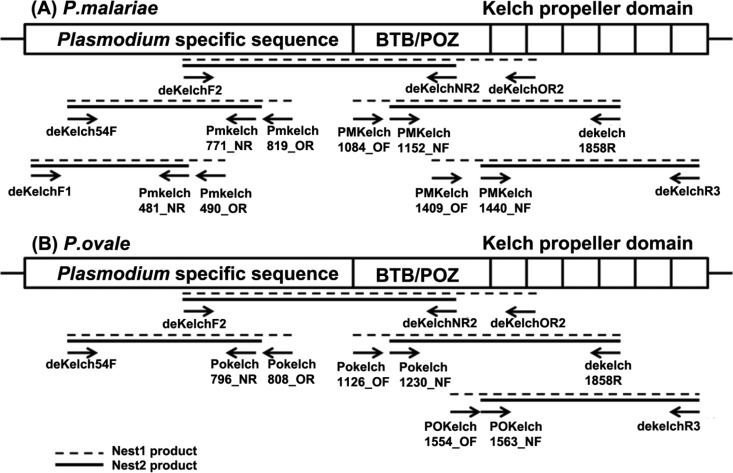
Schematic representation of the *kelch* propeller genes from P. malariae (A) and P. ovale (B). The positions of degenerate primers used for isolation of the *kelch* genes of these parasites are indicated. Dashed lines indicate the PCR products from the primary amplification reactions, and the thick lines indicate the PCR products obtained after the secondary amplification reactions.

For isolation of the P. malariae and P. ovale *kelch* genes, degenerate primers (Table 1 and Fig. 2) were designed to target the conserved region of the *kelch* genes from the other 3 human malaria parasites, i.e., P. falciparum (accession number PF3D7_1343700), P. vivax (accession number PVX_083080), and P. knowlesi (accession number PKH_121080).

**TABLE 1:** Degenerate oligonucleotide primers used for isolation of P. malariae and P. ovale kelch gene sequences

Primer	Sequence (5′ to 3′)^*a*^
deKelch_OR2	TCTCTYAAMCGATCATAYACCTCA
deKelch_F2	TATGARAAGAARATWATYGAAACG
deKelch_NR2	TTCAANACRGCACTTCCRAAATA
dekelch54F	SACGTAYGAWAGGGAATCTGG
dekelch1858R	CTGCNCCTGARCTTCTRGCTTC
deKelch_F1	ATGGARGRMGAAAAARTAAAANC
dekelchR3	CWATTAAAACGGARTGWCCAAATC
PMkelch810_OR	TTCTTTCATCATGTATTTTCTGC
PMkelch771_NR	TTCCTATTTTCAATTTCTTTGTATAAC
PMKelch1084_OF	GAAACATCCAGACATACGTTAACT
PMKelch1152_NF	TCATGTAACAAGAGATAAACAGGGTA
PMkelch490_OR	TCCTCGTTCGCATTTAAAGA
PMkelch481_NR	GCATTTAAAGATGCTGTTGC
PMkelch1409_OF	AAGCATATTTCGGTAGTGCAG
PMkelch1440_NF	TTTCTTGTATGTATTCGGAGGAAA
POkelch808_OR	TCTCTCATCATATAATTTTTGTTCTTC
POkelch796_NR	AATTTTTGTTCTTCTATAGCTTTTCT
POKelch1126_OF	TTCATTGAAAAATTATTAAGTGGTAGA
POKelch1230_NF	TTTTAAGAAACCCATTAACTGTACC
POkelch1554_OF	TGACCGTTTGAGAGATACATGG
POkelch1563_NF	TGAGAGATACATGGTTCGTTTCT

a The designations for degenerate sequences were used according to IUB nomenclature.

**FIG 3:**
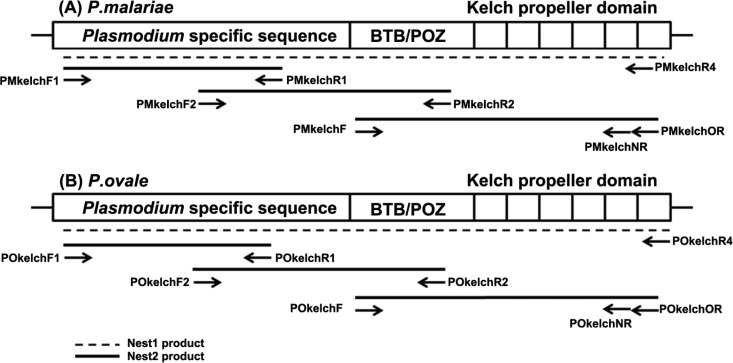
Diagrams of the *kelch* propeller genes from P. malariae (A) and P. ovale (B). The positions of specific oligonucleotide primers used for *Pmkelch* and *Pokelch* gene polymorphism investigation are indicated. Dashed lines indicate the PCR products from the primary amplification reactions, and the thick lines indicate the PCR products obtained after the secondary amplification reactions.

After partial sequences of the *kelch* gene were obtained from P. malariae and P. ovale, *Pmkelch*- and *Pokelch*-specific primers (Table 2 and Fig. 3) were subsequently designed to investigate polymorphisms in the gene. In this study, seminested and nested PCR approaches were used to increase the sensitivity and specificity of amplification. All primary PCRs and secondary reactions for DNA cloning were carried out in a total volume of 20 μl. Secondary reactions for direct DNA sequencing were performed in 100-μl mixtures. Reaction solutions contained 10 mM Tris-HCl, pH 8.3, 50 mM KCl, 2.5 mM MgCl_2_, 125 μM deoxynucleoside triphosphates (dNTPs), and 250 nM (each) primers for both the primary and secondary reactions, with 0.4 U of *Taq* polymerase (Invitrogen) included in each reaction mixture. In the primary reaction mixture, 1.5 μl of genomic DNA was used as the template; 2 μl of the products of the primary reaction was then used as the template for the secondary PCR. Cycling parameters for the PCRs were as follows: initial denaturation at 95°C for 5 min, followed by 30 cycles for primary PCR and 35 cycles for the secondary reaction (each cycle consisted of denaturing at 94°C for 1 min, annealing at 55°C for 2 min, and extension at 72°C for 2 min), with a final extension step at 72°C for 5 min. The temperature profile for the PCRs with P. malariae- and P. ovale-specific primers was as follows: initial denaturation at 95°C for 5 min, followed by 25 cycles for primary PCR and 30 cycles for the secondary reaction (each cycle consisted of denaturing at 94°C for 1 min, annealing at 55°C for 2 min [primary reaction] or 1 min [secondary reaction], and extension at 72°C for 2 min [primary reaction] or 1 min 30 s [secondary reaction]), with a final extension step at 72°C for 5 min. The PCR product size was estimated by comparison with a 100-bp DNA ladder. PCR products were cloned into the pGEM-T Easy vector (Promega) according to the manufacturer's instructions. The *Pmkelch*- and *Pokelch*-specific PCR products were purified using a FavorPrep gel/PCR purification kit (Farvogen, Taiwan) following the manufacturer's instructions.

**TABLE 2:** Specific oligonucleotide primers used for amplification of P. malariae and P. ovale kelch gene sequences

Primer^*a*^	Sequence (5′ to 3′)	Product size (bp)
PMkelchF1*	AAAAATAAAAGCCAACAGTATTTCAA	800
PMkelchR1	TGTCCAACTTCTTTCTTTCATCA	
PMkelchF2	AAATGCGAACGAGGAAAATG	750
PMkelchR2	TCACTCAAATCTTTTGGTATTGGA	
PMkelchR4*	TTAAAACGGAGTGACCAAATCTT	
PmKelch_F	TGATGAAGAAAGATTGAGATTCC	1,241
PmKelch_OR	TTGGAACAAGCAGAGAAGG	
PmKelch_NR	AAGCAGAGAAGGCCCAATTT	
POkelchF1*	GCGATGAGAAAAGTATGAGCAG	680
POkelchR1	TTCTTTCCATTTCTAATTCCTTCTT	
POkelchF2	CAACGTGCCGTTGAGAAATA	730
POkelchR2	CACGATCTAAAAATATCCTTCCTTG	
PokelchR4*	AAAACGGAGTGACCAAATCG	
PoKelch_F	TTTTGAAACTTCAAGACATACAC	1,066
PoKelch_OR	ATGGTCCTATCTGCCACTCG	
PoKelch_NR	GGGAACCAGTAAGGATGGTC	

a *, primers used in the primary reaction mixtures.

DNA sequencing was performed with both the forward and reverse strands to confirm the presence of polymorphisms. To ensure that the DNA sequences were from the P. malariae and P. ovale *kelch* genes, all DNA sequences were assessed with the Basic Local Alignment Search Tool (BLAST) at http://blast.ncbi.nlm.nih.gov/Blast.cgi. All sequences were then assembled, the *kelch* DNA and protein sequences were aligned with ClustalW software by use of Bioedit, and a phylogenetic analysis was constructed using the MEGA6 software program (Tokyo, Japan) (8). Phylogenetic relationships between the *kelch* genes of the different Plasmodium species were assessed by the neighbor-joining method. The Toxoplasma gondii kelch sequence (accession number XM_002365296) was used as the outgroup because small-subunit rRNA sequence data from other Apicomplexa have shown that Toxoplasma gondii is positioned prior to the stem that gives rise to Plasmodium spp. (9).

DNA sequences of all P. malariae and P. ovale isolates were analyzed for GC content and codon usage by use of the MEGA6 program and the Sequence Manipulation Suite package (SMS) (http://www.bioinformatics.org/sms2/index.html).

### Nucleotide sequence accession numbers.

The DNA sequences of *Pmkelch* and *Pokelch* have been submitted to GenBank under accession numbers KT792967 to KT792971.

## RESULTS

### Isolation of *Pmkelch* and *Pokelch*.

Several pairs of degenerate primers were designed based on the conserved region of the *kelch* genes from P. falciparum, P. vivax, and P. knowlesi, with the aim of amplifying overlapping *kelch* fragments. Five overlapping fragments of the P. malariae *kelch* gene and 4 fragments of the P. ovale wallikeri kelch gene (*PoWkelch*) were obtained, cloned, and sequenced (Fig. 2). The sizes of the assembled sequences of *Pmkelch* and *PoWkelch* were 2,087 and 2,063 bp, respectively. The *Pmkelch* gene encoded 695 amino acids, while *PoWkelch* encoded 687 amino acids. Four fragments of the P. ovale curtisi *kelch* gene (*PoCkelch*) were obtained by use of the *Pokelch*-specific primers, showing a 1,977-bp gene that encoded 658 amino acids of the partial P. ovale curtisi Kelch protein.

The coding sequences of the P. malariae, P. ovale wallikeri, and P. ovale curtisi *kelch* genes were aligned with other Plasmodium sp. *kelch* sequences (see Fig. S1 and S2 in the supplemental material) and compared between species. The proportions of amino acid homology between species are shown in Table 3, showing overall homologies of 82.7% to 98.5%, whereas compared to the human *kelch* gene, there was only 10.5% to 15% homology. It appeared that the BTB/POZ domain of the protein was the most highly conserved region (97.9 to 100%), whereas most polymorphisms were found in the Plasmodium-specific domain. Overall, *kelch* gene-encoded amino acid homologies were high between Plasmodium species, ranging from 93.3 to 99.6%. Homologies of both the P. malariae and P. ovale kelch genes to those of other Plasmodium species were similar to the homology in amino acids reported for a comparison between the P. falciparum and P. knowlesi kelch genes, which was reported as 88% for the overall *kelch* gene and 97% for the *kelch* propeller domain (5, 10).

**TABLE 3:** Comparison of homologies of malarial *kelch* genes

Species comparison^*a*^	% amino acid homology			
	Overall	Plasmodium-specific domain	BTB/POZ domain	Kelch domain
KEAP1 variant 1-PF	12.6	5.7	7.2	22.1
KEAP1 variant 1-PV	13.6	8.1	7.2	21.8
KEAP1 variant 1-PK	13.6	8.1	7.2	21.8
KEAP1 variant 1-PM	13.4	7.4	7.2	21.8
KEAP1 variant 1-POW	13.8	7.9	7.2	22.1
KEAP1 variant 1-POC	14.0	8.1	7.2	22.1
KEAP1 variant 2-PF	12.6	5.7	7.2	22.1
KEAP1 variant 2-PV	13.6	8.1	7.2	21.8
KEAP1 variant 2-PK	13.6	8.1	7.2	21.8
KEAP1 variant 2-PM	13.4	7.4	7.2	21.8
KEAP1 variant 2-POW	13.8	7.9	7.2	22.1
KEAP1 variant 2-POC	14.0	8.1	7.2	22.1
KLHL2 variant 1-PF	14.1	8.0	5.2	24.4
KLHL2 variant 1-PV	14.2	8.1	5.2	24.1
KLHL2 variant 1-PK	14.2	8.1	5.2	24.1
KLHL2 variant 1-PM	14.2	8.0	5.2	24.1
KLHL2 variant 1-POW	14.5	8.2	5.2	24.4
KLHL2 variant 1-POC	15.0	9.0	5.2	24.4
KLHL2 variant 2-PF	14.1	8.0	5.2	24.4
KLHL2 variant 2-PV	14.2	8.1	5.2	24.1
KLHL2 variant 2-PK	14.2	8.1	5.2	24.1
KLHL2 variant 2-PM	14.6	8.9	5.2	24.1
KLHL2 variant 2-POW	14.5	8.2	5.2	24.4
KLHL2 variant 2-POC	15.0	9.0	5.2	24.4
KLHL2 variant 3-PF	13.0	5.5	5.2	24.4
KLHL2 variant 3-PV	12.8	5.1	5.2	24.1
KLHL2 variant 3-PK	12.8	5.1	5.2	24.1
KLHL2 variant 3-PM	12.9	5.2	5.2	24.1
KLHL2 variant 3-POW	13.2	5.4	5.2	24.4
KLHL2 variant 3-POC	13.7	5.9	5.2	24.4
KLHL12 variant 1-PF	14.1	7.1	5.2	25.4
KLHL12 variant 1-PV	14.0	6.9	5.2	25.1
KLHL12 variant 1-PK	14.0	6.9	5.2	25.1
KLHL12 variant 1-PM	14.3	7.7	5.2	24.7
KLHL12 variant 1-POW	14.3	7.6	5.2	24.7
KLHL12 variant 1-POC	14.3	6.7	5.2	25.1
KLHL12 variant 2-PF	13.6	6.0	5.2	25.4
KLHL12 variant 2-PV	13.5	5.7	5.2	25.1
KLHL12 variant 2-PK	13.5	5.7	5.2	25.1
KLHL12 variant 2-PM	13.7	6.4	5.2	24.7
KLHL12 variant 2-POW	13.6	6.0	5.2	24.7
KLHL12 variant 2-POC	14.3	6.6	5.2	25.1
KLHL12 variant 3-PF	10.5	6.0	5.2	17.7
KLHL12 variant 3-PV	10.5	5.7	5.2	17.7
KLHL12 variant 3-PK	10.5	5.7	5.2	17.7
KLHL12 variant 3-PM	10.7	6.4	5.2	17.4
KLHL12 variant 3-POW	10.5	6.0	5.2	17.4
KLHL12 variant 3-POC	11.0	6.6	5.2	17.4
PF-PV	87.4	76.4	97.9	97.2
PF-PK	86.8	75.8	97.9	96.5
PF-PM	84.8	73.6	97.9	94.0
PF-POW	82.7	69.6	97.9	93.7
PF-POC	79.8	63.8	97.9	93.3
PV-PK	98.5	97.3	100.0	99.3
PV-PM	91.3	85.9	100.0	94.7
PV-POW	87.6	77.9	100.0	95.1
PV-POC	84.3	71.1	100.0	94.7
PK-PM	90.8	85.3	100.0	94.0
PK-POW	87.5	78.2	100.0	94.4
PK-POC	84.4	72.0	100.0	94.0
PM-POW	89.6	79.6	100.0	98.2
PM-POC	86.6	73.6	100.0	97.8
POW-POC	94.9	89.3	100.0	99.6

a KEAP1, human Kelch-like ECH-associated protein 1; KLHL, human Kelch-like family member; PF, P. falciparum; PV, P. vivax; PK, P. knowlesi; PM, P. malariae; POW, P. ovale wallikeri; POC, P. ovale curtisi.

Guanine-cytosine (GC) compositions of the different codons in the Plasmodium sp. *kelch* genes are shown in Table 4. P. malariae, P. ovale wallikeri, and P. ovale curtisi contained low GC contents, i.e., 28.9%, 27.9%, and 28.4%, respectively, in contrast to the high GC content found in the *kelch* genes of P. vivax and P. knowlesi. The GC contents of the *kelch* genes of P. malariae and P. ovale are similar to those of other P. malariae and P. ovale genes, e.g., *dhfr-ts* (accession numbers AY846634 and EU266606), *pppk-dhps* (accession number KJ400027), *18s rRNA* (accession numbers M54897 and L48987), *MSP1* (accession numbers FJ824669 and FJ824670), and Plasmepsin (accession number AF001210), within the range of 23.5 to 36.63% GC content. The *kelch* codon usage of each Plasmodium species is shown in Table 5. Codons containing only G and C were found in 1.15% (8/695 codons), 0.87% (6/687 codons), and 1.05% (7/658 codons) of codons in the P. malariae, P. ovale wallikeri, and P. ovale curtisi genes, respectively, which is similar to the case in P. falciparum (1.51% [11/727 codons]). The most prevalent amino acid codon in the *kelch* genes of P. malariae, P. ovale wallikeri, and P. ovale curtisi was an Asn codon (AAT), resulting in 63, 57, and 55 residues, respectively. Amino acid usages were similar between the different Plasmodium species.

**TABLE 4:** GC contents of malarial *kelch* genes

*kelch* gene domain and malaria species	GC content (%)			
	Overall	Codon position 1	Codon position 2	Codon position 3
Total gene				
P. falciparum	26.8	36.0	29.6	14.7
P. vivax	38.9	38.6	30.4	47.5
P. knowlesi	35.2	37.3	30.3	38.1
P. malariae	28.9	34.5	30.1	19.1
P. ovale wallikeri	27.9	35.9	29.7	21.2
P. ovale curtisi	28.4	36.3	29.3	19.7
Plasmodium-specific domain				
P. falciparum	21.8	30.4	22.0	13.0
P. vivax	34.1	33.5	23.0	45.9
P. knowlesi	30.5	30.8	22.7	38.1
P. malariae	24.9	28.3	22.5	23.9
P. ovale wallikeri	24.6	29.0	21.5	23.3
P. ovale curtisi	23.7	30.1	19.7	21.4
BTB/POZ domain				
P. falciparum	28.9	37.1	34.0	15.5
P. vivax	39.9	41.2	34.0	44.3
P. knowlesi	37.1	42.3	34.0	35.1
P. malariae	28.5	38.1	34.0	13.4
P. ovale wallikeri	26.5	39.2	34.0	6.2
P. ovale curtisi	27.8	38.1	34.0	11.3
Kelch domain				
P. falciparum	32.0	42.5	37.2	16.5
P. vivax	44.0	43.5	37.9	50.5
P. knowlesi	40.1	43.2	37.9	39.3
P. malariae	31.2	40.5	37.7	15.4
P. ovale wallikeri	34.9	42.7	37.7	24.2
P. ovale curtisi	33.6	42.1	37.7	21.0

**TABLE 5:** Codon usage in the malarial *kelch* gene

Codon	Amino acid	No. of occurrences^*a*^					
		PF	PV	PK	PM	POW	POC
AAG	Lys	5	27	23	9	10	11
AAA	Lys	57	36	39	51	49	45
AAT	Asn	81	44	51	63	57	55
AAC	Asn	8	31	25	13	21	20
ACG	Thr	4	9	10	6	5	5
ACA	Thr	12	6	7	15	10	11
ACT	Thr	10	5	8	8	9	6
ACC	Thr	5	5	1	1	2	2
AGG	Arg	3	11	8	1	1	1
AGA	Arg	21	12	18	23	24	24
AGT	Ser	21	10	17	23	23	18
AGC	Ser	4	19	12	5	6	4
ATA	Ile	18	18	19	23	26	26
ATT	Ile	29	15	18	24	15	15
ATC	Ile	2	15	12	2	3	3
ATG	Met	18	21	21	19	16	15
CAG	Gln	2	8	7	5	4	2
CAA	Gln	14	8	9	11	13	15
CAT	His	9	3	2	6	4	4
CAC	His	1	5	6	1	3	3
CCG	Pro	2	3	2	0	1	1
CCA	Pro	10	7	8	12	9	11
CCT	Pro	4	2	3	4	8	6
CCC	Pro	2	7	6	1	1	0
CGG	Arg	0	1	2	2	0	0
CGA	Arg	3	5	2	1	2	2
CGT	Arg	4	0	0	3	3	3
CGC	Arg	0	1	1	0	0	0
CTG	Leu	1	9	6	1	2	2
CTA	Leu	5	10	10	1	7	5
CTT	Leu	6	4	5	2	5	5
CTC	Leu	0	7	5	1	1	1
GAG	Glu	10	19	13	9	9	6
GAA	Glu	48	41	47	51	47	46
GAT	Asp	46	29	31	37	38	38
GAC	Asp	0	16	15	7	8	5
GCG	Ala	0	2	3	1	1	1
GCA	Ala	11	8	8	17	9	10
GCT	Ala	11	4	7	5	10	9
GCC	Ala	3	10	5	1	1	2
GGG	Gly	1	13	9	2	2	2
GGA	Gly	17	10	11	11	14	13
GGT	Gly	17	7	11	17	16	16
GGC	Gly	3	7	4	1	0	1
GTG	Val	2	7	4	2	3	1
GTA	Val	16	4	6	12	8	11
GTT	Val	13	9	12	14	15	17
GTC	Val	1	9	6	2	2	0
TAG	Stop	0	0	0	0	0	0
TAA	Stop	1	0	0	0	0	0
TAT	Tyr	25	17	19	24	22	23
TAC	Tyr	3	10	8	2	5	4
TCG	Ser	3	9	7	4	5	5
TCA	Ser	13	5	7	12	8	7
TCT	Ser	14	6	9	9	13	11
TCC	Ser	3	18	14	10	7	7
TGA	Stop	0	1	1	0	0	0
TGG	Trp	7	7	7	7	7	7
TGT	Cys	6	5	6	7	7	7
TGC	Cys	1	2	2	0	0	0
TTG	Leu	7	14	14	10	12	13
TTA	Leu	46	19	22	41	38	36
TTT	Phe	32	24	28	36	32	33
TTC	Phe	6	17	14	7	8	6

a PF, P. falciparum; PV, P. vivax; PK, P. knowlesi; PM, P. malariae; POW, P. ovale wallikeri; POC, P. ovale curtisi.

To exclude the possibility of interspecies cross-reactivity in the isolation of *Pmkelch* and *Pokelch* in our experiments, we first tested the specificity of amplification by applying the specific primers targeting the *kelch* genes of P. malariae and P. ovale to samples from patients with monoinfections with P. falciparum (*n* = 20) and P. vivax (*n* = 20), as well as to samples from healthy volunteers (*n* = 20). Parasitemias varied between 4,000 parasites/μl and 35,000 parasites/μl. None of the P. falciparum, P. vivax, or control samples showed any DNA amplification, confirming the P. malariae and P. ovale species-specific amplification obtained using these primers. Second, the specificity of amplification was demonstrated by showing that the 4 amplified fragments of *Pmkelch* were from the same gene. Specific *Pmkelch* primers (PMkelchF1 and PMkelchR4) (Table 2) which can bind to the 5′ and 3′ ends of the gene were used to amplify the 2-kb sequence of this gene. The 2-kb *Pmkelch* fragment was then cloned into a pGEM vector and sequenced with all 4 forward *Pmkelch* primers. The same approach was used to determine the specificity of amplification of *Pokelch*. The assembled DNA sequences obtained by primer walking, with plasmid replication in Escherichia coli, showed that all 4 fragments were from the *Pmkelch* gene or the *Pokelch* gene. From the plasmid amplification, 3 SNPs (F195S, K649E, and S659T) were observed in the *Pmkelch* gene, and 6 SNPs (N93D, K104I, N154S, E235G, D531Y, and A599T) were observed in the *Pokelch* gene. However, by direct sequencing of the PCR products, these 9 SNPs appeared to all be false-positive findings, which is a known potential artifact of this DNA cloning process. Third, all DNA fragments were assessed by BLAST searches and in multiple-sequence alignments with genes from other Plasmodium species. The percentages of homology of the isolated P. malariae and P. ovale sequences are in the range that can differentiate Plasmodium species based on each domain.

The *kelch* orthologues of P. ovale wallikeri and P. ovale curtisi were isolated by using the same primer set and approach. The differences between these two species were tested and confirmed with other protocols (3, 11). All of the *P. ovale* isolates were from single infections. Therefore, the isolated *kelch* genes were clearly derived from P. ovale wallikeri and P. ovale curtisi, with the same range of homology (89.3 to 94.9%) as that in a previous study (3).

### Analysis of polymorphisms in *Pmkelch* and *Pokelch*.

To determine the presence of polymorphisms in the *Pmkelch* and *Pokelch* genes in samples from Thailand, nested PCR was performed following the protocol presented in Materials and Methods (Fig. 3). All PCR products were purified and processed for DNA sequencing. The sequences of the 13 isolates of P. malariae were aligned, and only 1 isolate, PM048, had a nonsynonymous mutation, at nucleotide position 1600 (A to T), resulting in the amino acid change P533L. The sequences of the 5 isolates of P. ovale (4 P. ovale wallikeri and 1 P. ovale curtisi isolate) were also aligned, and only 1 P. ovale wallikeri isolate, PoW20, had a nonsynonymous mutation, A411G, encoding the amino acid change K137R (Table 6).

**TABLE 6:** Nonsynonymous mutations observed for P. malariae, P. ovale, and other human malaria parasites

Isolate no.	Isolate or accession no.^*a*^	Organism	Amino acid residue at position corresponding to nonsynonymous mutation:	
			K137R	P533L
	PF3D7_1343700	P. falciparum	K168	P553
	PVX_083080	P. vivax	K152	P539
	PKH_121080	P. knowlesi	K152	P539
1	PM1A	P. malariae		P
2	PM2	P. malariae		P
3	PMS	P. malariae		P
4	PM1381	P. malariae		P
5	PM17	P. malariae		P
6	PM18	P. malariae		P
7	PM048	P. malariae		L
8	PM1S	P. malariae		P
9	PM4	P. malariae		P
10	PM2848	P. malariae		P
11	PM454	P. malariae		P
12	PM5	P. malariae		P
13	PM1454	P. malariae		P
14	PoW1	P. ovale wallikeri	K	
15	PoW21	P. ovale wallikeri	K	
16	PoW23	P. ovale wallikeri	K	
17	PoC13	P. ovale curtisi	K	
18	PoW20	P. ovale wallikeri	R	

a Accession numbers for PM2, PM048, PoW1, PoC13, and PoW20 are KT792967 to KT792971, respectively.

**FIG 4:**
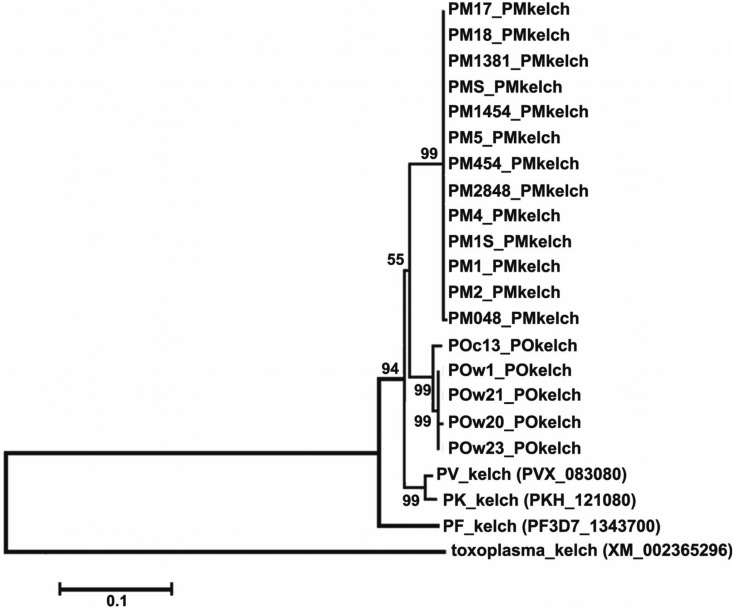
Phylogenetic relationships among Plasmodium sp. *kelch* genes. Toxoplasma gondii was used as an outgroup to root the tree.

Phylogenetic analysis of the *kelch* genes of all human Plasmodium species is shown in Fig. 4. It was shown that a group of 13 P. malariae strains formed a single branch. Five isolates of P. ovale formed 2 branches, dividing the P. ovale strains into P. ovale curtisi and P. ovale wallikeri. The phylogenetic tree reveals the clear clustering of each Plasmodium species, with the newly isolated strains (13 P. malariae, 4 P. ovale wallikeri, and 1 P. ovale curtisi strain) located according to their relationships.

## DISCUSSION

This study isolates and explores, for the first time, the *kelch* orthologue genes from P. malariae, P. ovale curtisi, and P. ovale wallikeri. Analysis of the *kelch* genes from 13 P. malariae isolates from Thailand showed limited polymorphism, with only one nonsynonymous mutation, P533L, found in a single isolate. This mutation is equivalent to the previously reported P553L mutation in *Pfkelch* (12–14). The observed mutation can be linked to artemisinin drug pressure, but it may also represent the natural background variability of the gene (15). Whether the P553L mutation affects Kelch 13 functionally will need further study. Moreover, since the sample size of the current study was relatively small, further monitoring for polymorphisms of the *kelch* gene in P. malariae and P. ovale is warranted. Studying *kelch* in these other Plasmodium species will be valuable for our further understanding of this gene and its polymorphisms in all human malaria parasites.

There were 5 samples containing P. ovale included in this study: 4 P. ovale wallikeri and 1 P. ovale curtisi isolate. In the P. ovale wallikeri strains, only one nonsynonymous mutation in *kelch*, K137R, was found. The K137R mutation is located in the Plasmodium-specific domain. Multiple-sequence alignment of Plasmodium Kelch amino acids showed that there are a number of polymorphisms within the Plasmodium-specific domain. Collection of more *Pokelch* genes from more samples would provide insight into the specific sequence for each Plasmodium species.

Genetic analysis of nucleotide composition within the *kelch* genes of all human Plasmodium species showed similar trends in GC content and codon usage. Overall, GC contents were similar, ranging from 26.8 to 35.2% (Table 4), which is slightly different from those of other genes. For comparison, the GC content of the *pppk-dhps* gene of P. malariae is similar to that for P. falciparum (23.5 to 27.8%), whereas the content in P. vivax is slightly higher (43.2%) (16). The homologies of the *kelch* genes of all human malarial species showed that P. falciparum separates from the other species. Multiple-sequence alignment of amino acids within the Kelch Plasmodium-specific domain (see Fig. S1 and S2 in the supplemental material) clearly showed the conservation of amino acid sequences among P. vivax, P. malariae, P. ovale, and P. knowlesi, whereas P. falciparum showed an alternative codon. The same pattern was found for non-Plasmodium-specific domains. This characteristic facilitated the generation of the phylogenetic tree topology (Fig. 4), which showed that P. falciparum was first separated as a single branch distant from the other Plasmodium species. *kelch* gene homology confirmed the close genetic relationship between P. ovale wallikeri and P. ovale curtisi (94.9%), in line with previous reports comparing other genes between these species (3, 11). This is in accordance with the presence of sympatric P. ovale wallikeri and P. ovale curtisi in Thailand.

Phylogenetic analysis showed the close relatedness of *kelch* gene sequences between Plasmodium species infecting humans, but it also showed clear clustering patterns for the *Pmkelch* and *Pokelch* genes defining the different species. This shows that the isolated *kelch* genes from both P. malariae and P. ovale are species specific and are not variants of the gene from any other human Plasmodium species. This separation of clades according to Plasmodium species is consistent with patterns described previously for other genes, such as *msp1* (17).

In conclusion, this study is the first report of the isolation and analysis of the *kelch*-orthologous genes of P. malariae, P. ovale wallikeri, and P. ovale curtisi. Only a single point mutation in *kelch* was observed among 13 P. malariae isolates. Its functional consequences for artemisinin sensitivity in P. malariae and P. ovale remain to be elucidated. To obtain a more complete picture of the genetic epidemiology of the artemisinin resistance-associated *kelch* gene in all human malaria species, *kelch* orthologue gene polymorphisms will need to be studied in a larger sample of P. malariae, P. ovale wallikeri, and P. ovale curtisi strains.

## Supplementary Material

Supplemental material
