# Supplementary material for: Limited Polymorphism of the Kelch Propeller Domain in Plasmodium malariae and P. ovale Isolates from Thailand
Source: Antimicrob Agents Chemother. 2016 Jun 20;60(7):4055–62. doi: 10.1128/AAC.00138-16 (PMC4914644; doi:10.1128/AAC.00138-16)
Supplement: Supplemental material [file supp_60_7_4055__index.html]

Limited Polymorphism of the Kelch Propeller Domain in Plasmodium malariae and P. ovale Isolates from Thailand — Supplemental material 

# Limited Polymorphism of the Kelch Propeller Domain in Plasmodium malariae and P. ovale Isolates from Thailand

## Supplemental material

- Supplemental file 1 -

  Figures S1 and S2

  PDF, 45K
