# Supplementary material for: Limited Polymorphism of the Kelch Propeller Domain in Plasmodium malariae and P. ovale Isolates from Thailand
Source: Antimicrob Agents Chemother. 2016 Jun 20;60(7):4055–62. doi: 10.1128/AAC.00138-16 (PMC4914644; doi:10.1128/AAC.00138-16)
Supplement: Supplemental material [file AAC.00138-16_zac007165292so1.pdf]

```
PF_kelch(PF3D7_1343700)     MEGEKVKTKANSISNFSMTYDRESGGNSNSDDKSGSSSENDSNSFMNLTSDKNEKTENNS    60
PM2_PMkelch                 ----------NSISNFSVTYDRESGVNSNSDDRSESSSENESNSFMNMTSDKNEKTENNS    50
PM1_PMkelch                 ---------------------------SNSDDRSESSSENESNSFMNMTSDKNEKTENNS    33
PM1S_PMkelch                ---------------------------SNSDDRSESSSENESNSFMNMTSDKNEKTENNS    33
PM4_PMkelch                 ---------------------------SNSDDRSESSSENESNSFMNMTSDKNEKTENNS    33
PM2848_PMkelch              ---------------------------SNSDDRSESSSENESNSFMNMTSDKNEKTENNS    33
PM454_PMkelch               ---------------------------SNSDDRSESSSENESNSFMNMTSDKNEKTENNS    33
PM5_PMkelch                 ---------------------------SNSDDRSESSSENESNSFMNMTSDKNEKTENNS    33
PM1454_PMkelch              ---------------------------SNSDDRSESSSENESNSFMNMTSDKNEKTENNS    33
PMS_PMkelch                 ----------------------------------------ESNSFMNMTSDKNEKTENNS    20
PM1381_PMkelch              ----------------------------------------ESNSFMNMTSDKNEKTENNS    20
PM17_PMkelch                ----------------------------------------ESNSFMNMTSDKNEKTENNS    20
PM18_PMkelch                ----------------------------------------ESNSFMNMTSDKNEKTENNS    20
PM048_PMkelch               ----------------------------------------ESNSFMNMTSDKNEKTENNS    20
PV_kelch(PVX_083080)        MEGEKI--KSNSISNFSVTYERESGANSNSDDKSVSSSENESNSFMNLTSDKNEKTENNS    58
PK_kelch(PKH_121080)        MEDEKI--KSNSISNFSVTYERESGANSNSDDKSVSSSENESNSFMNLTSDKNEKTENNS    58

PF_kelch(PF3D7_1343700)     FLLNNSSYGNVKDSLLESIDMSVLDSNFDSKKDFLPSNLSRTFNNMSKDNIGNKYLNKLL    120
PM2_PMkelch                 FALNNSSFVNMKDSLLESIDLSVLDSNFDTKKDFLPSNFSKNFNNLSKENISNKYLNKFL    110
PM1_PMkelch                 FALNNSSFVNMKDSLLESIDLSVLDSNFDTKKDFLPSNFSKNFNNLSKENISNKYLNKFL    93
PM1S_PMkelch                FALNNSSFVNMKDSLLESIDLSVLDSNFDTKKDFLPSNFSKNFNNLSKENISNKYLNKFL    93
PM4_PMkelch                 FALNNSSFVNMKDSLLESIDLSVLDSNFDTKKDFLPSNFSKNFNNLSKENISNKYLNKFL    93
PM2848_PMkelch              FALNNSSFVNMKDSLLESIDLSVLDSNFDTKKDFLPSNFSKNFNNLSKENISNKYLNKFL    93
PM454_PMkelch               FALNNSSFVNMKDSLLESIDLSVLDSNFDTKKDFLPSNFSKNFNNLSKENISNKYLNKFL    93
PM5_PMkelch                 FALNNSSFVNMKDSLLESIDLSVLDSNFDTKKDFLPSNFSKNFNNLSKENISNKYLNKFL    93
PM1454_PMkelch              FALNNSSFVNMKDSLLESIDLSVLDSNFDTKKDFLPSNFSKNFNNLSKENISNKYLNKFL    93
PMS_PMkelch                 FALNNSSFVNMKDSLLESIDLSVLDSNFDTKKDFLPSNFSKNFNNLSKENISNKYLNKFL    80
PM1381_PMkelch              FALNNSSFVNMKDSLLESIDLSVLDSNFDTKKDFLPSNFSKNFNNLSKENISNKYLNKFL    80
PM17_PMkelch                FALNNSSFVNMKDSLLESIDLSVLDSNFDTKKDFLPSNFSKNFNNLSKENISNKYLNKFL    80
PM18_PMkelch                FALNNSSFVNMKDSLLESIDLSVLDSNFDTKKDFLPSNFSKNFNNLSKENISNKYLNKFL    80
PM048_PMkelch               FALNNSSFVNMKDSLLESIDLSVLDSNFDTKKDFLPSNFSKNFNNLSKENISNKYLNKFL    80
PV_kelch(PVX_083080)        FILNNSSFANMKDSLLESIDLSVLDSNFDSKKDFLPSNLSKNFNNLSKENLGNKYLNKLL    118
PK_kelch(PKH_121080)        FILNNSSFANMKDSFLESIDLSILDSNFDSKKDFLPSNLSKNFNNLSKENLGNKYLNKLL    118

PF_kelch(PF3D7_1343700)     NKKKDTITNENNNINHNNNNNNNLTANNITNNLINNNMNSPSIMNTNKKENFLDAAN---L    177
PM2_PMkelch                 NKSDSMFMSKSKDMNLTDASNN--------------NVNISVKNNTKKEIFMDAATASLN    156
PM1_PMkelch                 NKSDSMFMSKSKDMNLTDASNN--------------NVNISVKNNTKKEIFMDAATASLN    139
PM1S_PMkelch                NKSDSMFMSKSKDMNLTDASNN--------------NVNISVKNNTKKEIFMDAATASLN    139
PM4_PMkelch                 NKSDSMFMSKSKDMNLTDASNN--------------NVNISVKNNTKKEIFMDAATASLN    139
PM2848_PMkelch              NKSDSMFMSKSKDMNLTDASNN--------------NVNISVKNNTKKEIFMDAATASLN    139
PM454_PMkelch               NKSDSMFMSKSKDMNLTDASNN--------------NVNISVKNNTKKEIFMDAATASLN    139
PM5_PMkelch                 NKSDSMFMSKSKDMNLTDASNN--------------NVNISVKNNTKKEIFMDAATASLN    139
PM1454_PMkelch              NKSDSMFMSKSKDMNLTDASNN--------------NVNISVKNNTKKEIFMDAATASLN    139
PMS_PMkelch                 NKSDSMFMSKSKDMNLTDASNN--------------NVNISVKNNTKKEIFMDAATASLN    126
PM1381_PMkelch              NKSDSMFMSKSKDMNLTDASNN--------------NVNISVKNNTKKEIFMDAATASLN    126
PM17_PMkelch                NKSDSMFMSKSKDMNLTDASNN--------------NVNISVKNNTKKEIFMDAATASLN    126
PM18_PMkelch                NKSDSMFMSKSKDMNLTDASNN--------------NVNISVKNNTKKEIFMDAATASLN    126
PM048_PMkelch               NKSDSMFMSKSKDMNLTDASNN--------------NVNISVKNNTKKEIFMDAATASLN    126
PV_kelch(PVX_083080)        NKSDSMFMSKGKDMNLMENNLG--------------SNNLPVKSSNKKEGFMDSST-PIN    163
PK_kelch(PKH_121080)        NKSDSLFMSKNKDMNLMDNNMG--------------SNNLPVKSSNRKEGFMDSST-PIN    163

PF_kelch(PF3D7_1343700)     INDDSGLNNLKKFSTV-NNVNDTYEKKIIETELSDASDFENMVGDLRITFINWLKKTQMN    236
PM2_PMkelch                 ANEENAMNNLKKFTNTNNNINDTYEKKIIETELSDSSDFENMVGDLRITFINWLKKTQMN    216
PM1_PMkelch                 ANEENAMNNLKKFTNTNNNINDTYEKKIIETELSDSSDFENMVGDLRITFINWLKKTQMN    199
PM1S_PMkelch                ANEENAMNNLKKFTNTNNNINDTYEKKIIETELSDSSDFENMVGDLRITFINWLKKTQMN    199
PM4_PMkelch                 ANEENAMNNLKKFTNTNNNINDTYEKKIIETELSDSSDFENMVGDLRITFINWLKKTQMN    199
PM2848_PMkelch              ANEENAMNNLKKFTNTNNNINDTYEKKIIETELSDSSDFENMVGDLRITFINWLKKTQMN    199
PM454_PMkelch               ANEENAMNNLKKFTNTNNNINDTYEKKIIETELSDSSDFENMVGDLRITFINWLKKTQMN    199
PM5_PMkelch                 ANEENAMNNLKKFTNTNNNINDTYEKKIIETELSDSSDFENMVGDLRITFINWLKKTQMN    199
PM1454_PMkelch              ANEENAMNNLKKFTNTNNNINDTYEKKIIETELSDSSDFENMVGDLRITFINWLKKTQMN    199
PMS_PMkelch                 ANEENAMNNLKKFTNTNNNINDTYEKKIIETELSDSSDFENMVGDLRITFINWLKKTQMN    186
PM1381_PMkelch              ANEENAMNNLKKFTNTNNNINDTYEKKIIETELSDSSDFENMVGDLRITFINWLKKTQMN    186
PM17_PMkelch                ANEENAMNNLKKFTNTNNNINDTYEKKIIETELSDSSDFENMVGDLRITFINWLKKTQMN    186
PM18_PMkelch                ANEENAMNNLKKFTNTNNNINDTYEKKIIETELSDSSDFENMVGDLRITFINWLKKTQMN    186
PM048_PMkelch               ANEENAMNNLKKFTNTNNNINDTYEKKIIETELSDSSDFENMVGDLRITFINWLKKTQMN    186
PV_kelch(PVX_083080)        ANEDNAMNNLKKYSNA-NNINDTYEKKIIETELSDSSDFENMVGDLRITFINWLKKTQMN    222
PK_kelch(PKH_121080)        ANEDNAMNNLKKYSNT-NNINDTYEKKIIETELSDSSDFENMVGDLRITFINWLKKTQMN    222

PF_kelch(PF3D7_1343700)     FIREKDKLFKDKKELEMERVRLYKELENRKNIEEQKLHDERKKLDIDISNGYKQIKKEKE    296
PM2_PMkelch                 FIREKDKLFKDKKELEMERIRLYKEIENRKIIEEQKIHDERKKLDIDISNGYKQIKKEKE    276
PM1_PMkelch                 FIREKDKLFKDKKELEMERIRLYKEIENRKIIEEQKIHDERKKLDIDISNGYKQIKKEKE    259
PM1S_PMkelch                FIREKDKLFKDKKELEMERIRLYKEIENRKIIEEQKIHDERKKLDIDISNGYKQIKKEKE    259
PM4_PMkelch                 FIREKDKLFKDKKELEMERIRLYKEIENRKIIEEQKIHDERKKLDIDISNGYKQIKKEKE    259
```

```
PM2848_PMkelch            FIREKDKLFKDKKELEMERIRLYKEIENRKIIEEQKIHDERKKLDIDISNGYKQIKKEKE    259
PM454_PMkelch             FIREKDKLFKDKKELEMERIRLYKEIENRKIIEEQKIHDERKKLDIDISNGYKQIKKEKE    259
PM5_PMkelch               FIREKDKLFKDKKELEMERIRLYKEIENRKIIEEQKIHDERKKLDIDISNGYKQIKKEKE    259
PM1454_PMkelch            FIREKDKLFKDKKELEMERIRLYKEIENRKIIEEQKIHDERKKLDIDISNGYKQIKKEKE    259
PMS_PMkelch               FIREKDKLFKDKKELEMERIRLYKEIENRKIIEEQKIHDERKKLDIDISNGYKQIKKEKE    246
PM1381_PMkelch            FIREKDKLFKDKKELEMERIRLYKEIENRKIIEEQKIHDERKKLDIDISNGYKQIKKEKE    246
PM17_PMkelch              FIREKDKLFKDKKELEMERIRLYKEIENRKIIEEQKIHDERKKLDIDISNGYKQIKKEKE    246
PM18_PMkelch              FIREKDKLFKDKKELEMERIRLYKEIENRKIIEEQKIHDERKKLDIDISNGYKQIKKEKE    246
PM048_PMkelch             FIREKDKLFKDKKELEMERIRLYKEIENRKIIEEQKIHDERKKLDIDISNGYKQIKKEKE    246
PV_kelch(PVX_083080)      FIREKDKLFKDKKELEMERIRLYKEIENRKSIEEQKLHDERKKLDIDISNGYKQIKKEKE    282
PK_kelch(PKH_121080)      FIREKDKLFKDKKELEMERIRLYKEIENRKSIEEQKLHDERKKLDIDISNGYKQIKKEKE    282


PF_kelch(PF3D7_1343700)   EHRKRFDEERLRFLQEIDKIKLVLYLEKEKYYQEYKNFENDKKKIVDANIATETMIDINV    356
PM2_PMkelch               EHRKRFDEERLRFLQEIDKIKLVLYLEKEKYFQEYKNFENDKKKIVDANIATETMIDINV    336
PM1_PMkelch               EHRKRFDEERLRFLQEIDKIKLVLYLEKEKYFQEYKNFENDKKKIVDANIATETMIDINV    319
PM1S_PMkelch              EHRKRFDEERLRFLQEIDKIKLVLYLEKEKYFQEYKNFENDKKKIVDANIATETMIDINV    319
PM4_PMkelch               EHRKRFDEERLRFLQEIDKIKLVLYLEKEKYFQEYKNFENDKKKIVDANIATETMIDINV    319
PM2848_PMkelch            EHRKRFDEERLRFLQEIDKIKLVLYLEKEKYFQEYKNFENDKKKIVDANIATETMIDINV    319
PM454_PMkelch             EHRKRFDEERLRFLQEIDKIKLVLYLEKEKYFQEYKNFENDKKKIVDANIATETMIDINV    319
PM5_PMkelch               EHRKRFDEERLRFLQEIDKIKLVLYLEKEKYFQEYKNFENDKKKIVDANIATETMIDINV    319
PM1454_PMkelch            EHRKRFDEERLRFLQEIDKIKLVLYLEKEKYFQEYKNFENDKKKIVDANIATETMIDINV    319
PMS_PMkelch               EHRKRFDEERLRFLQEIDKIKLVLYLEKEKYFQEYKNFENDKKKIVDANIATETMIDINV    306
PM1381_PMkelch            EHRKRFDEERLRFLQEIDKIKLVLYLEKEKYFQEYKNFENDKKKIVDANIATETMIDINV    306
PM17_PMkelch              EHRKRFDEERLRFLQEIDKIKLVLYLEKEKYFQEYKNFENDKKKIVDANIATETMIDINV    306
PM18_PMkelch              EHRKRFDEERLRFLQEIDKIKLVLYLEKEKYFQEYKNFENDKKKIVDANIATETMIDINV    306
PM048_PMkelch             EHRKRFDEERLRFLQEIDKIKLVLYLEKEKYFQEYKNFENDKKKIVDANIATETMIDINV    306
PV_kelch(PVX_083080)      EHRKRFDEERLRFLQEIDKIKLVLYLEKEKYFQEYKNFENDKKKIVDANIATETMIDINV    342
PK_kelch(PKH_121080)      EHRKRFDEERLRFLQEIDKIKLVLYLEKEKYFQEYKNFENDKKKIVDANIATETMIDINV    342

PF_kelch(PF3D7_1343700)   GGAIFETSRHTLTQQKDSFIEKLLSGRHHVTRDKQGRIFLDRDSELFRIILNFLRNPLTI    416
PM2_PMkelch               GGAIFETSRHTLTQQKDSFIEKLLSGRYHVTRDKQGRIFLDRDSELFRIILNFLRNPLTV    396
PM1_PMkelch               GGAIFETSRHTLTQQKDSFIEKLLSGRYHVTRDKQGRIFLDRDSELFRIILNFLRNPLTV    379
PM1S_PMkelch              GGAIFETSRHTLTQQKDSFIEKLLSGRYHVTRDKQGRIFLDRDSELFRIILNFLRNPLTV    379
PM4_PMkelch               GGAIFETSRHTLTQQKDSFIEKLLSGRYHVTRDKQGRIFLDRDSELFRIILNFLRNPLTV    379
PM2848_PMkelch            GGAIFETSRHTLTQQKDSFIEKLLSGRYHVTRDKQGRIFLDRDSELFRIILNFLRNPLTV    379
PM454_PMkelch             GGAIFETSRHTLTQQKDSFIEKLLSGRYHVTRDKQGRIFLDRDSELFRIILNFLRNPLTV    379
PM5_PMkelch               GGAIFETSRHTLTQQKDSFIEKLLSGRYHVTRDKQGRIFLDRDSELFRIILNFLRNPLTV    379
PM1454_PMkelch            GGAIFETSRHTLTQQKDSFIEKLLSGRYHVTRDKQGRIFLDRDSELFRIILNFLRNPLTV    379
PMS_PMkelch               GGAIFETSRHTLTQQKDSFIEKLLSGRYHVTRDKQGRIFLDRDSELFRIILNFLRNPLTV    366
PM1381_PMkelch            GGAIFETSRHTLTQQKDSFIEKLLSGRYHVTRDKQGRIFLDRDSELFRIILNFLRNPLTV    366
PM17_PMkelch              GGAIFETSRHTLTQQKDSFIEKLLSGRYHVTRDKQGRIFLDRDSELFRIILNFLRNPLTV    366
PM18_PMkelch              GGAIFETSRHTLTQQKDSFIEKLLSGRYHVTRDKQGRIFLDRDSELFRIILNFLRNPLTV    366
PM048_PMkelch             GGAIFETSRHTLTQQKDSFIEKLLSGRYHVTRDKQGRIFLDRDSELFRIILNFLRNPLTV    366
PV_kelch(PVX_083080)      GGAIFETSRHTLTQQKDSFIEKLLSGRYHVTRDKQGRIFLDRDSELFRIILNFLRNPLTV    402
PK_kelch(PKH_121080)      GGAIFETSRHTLTQQKDSFIEKLLSGRYHVTRDKQGRIFLDRDSELFRIILNFLRNPLTV    402

PF_kelch(PF3D7_1343700)   PIPKDLSESEALLKEAEFYGIKFLPFPLVFCIGGFDGVEYLNSMELLDISQQCWRMCTPM    476
PM2_PMkelch               PIPKDLSESEALLKEAEFYGIKFLPFPLVFCIGGFDGVEYLNSMELLDISQQCWRMCTPM    456
PM1_PMkelch               PIPKDLSESEALLKEAEFYGIKFLPFPLVFCIGGFDGVEYLNSMELLDISQQCWRMCTPM    439
PM1S_PMkelch              PIPKDLSESEALLKEAEFYGIKFLPFPLVFCIGGFDGVEYLNSMELLDISQQCWRMCTPM    439
PM4_PMkelch               PIPKDLSESEALLKEAEFYGIKFLPFPLVFCIGGFDGVEYLNSMELLDISQQCWRMCTPM    439
PM2848_PMkelch            PIPKDLSESEALLKEAEFYGIKFLPFPLVFCIGGFDGVEYLNSMELLDISQQCWRMCTPM    439
PM454_PMkelch             PIPKDLSESEALLKEAEFYGIKFLPFPLVFCIGGFDGVEYLNSMELLDISQQCWRMCTPM    439
PM5_PMkelch               PIPKDLSESEALLKEAEFYGIKFLPFPLVFCIGGFDGVEYLNSMELLDISQQCWRMCTPM    439
PM1454_PMkelch            PIPKDLSESEALLKEAEFYGIKFLPFPLVFCIGGFDGVEYLNSMELLDISQQCWRMCTPM    439
PMS_PMkelch               PIPKDLSESEALLKEAEFYGIKFLPFPLVFCIGGFDGVEYLNSMELLDISQQCWRMCTPM    426
PM1381_PMkelch            PIPKDLSESEALLKEAEFYGIKFLPFPLVFCIGGFDGVEYLNSMELLDISQQCWRMCTPM    426
PM17_PMkelch              PIPKDLSESEALLKEAEFYGIKFLPFPLVFCIGGFDGVEYLNSMELLDISQQCWRMCTPM    426
PM18_PMkelch              PIPKDLSESEALLKEAEFYGIKFLPFPLVFCIGGFDGVEYLNSMELLDISQQCWRMCTPM    426
PM048_PMkelch             PIPKDLSESEALLKEAEFYGIKFLPFPLVFCIGGFDGVEYLNSMELLDISQQCWRMCTPM    426
PV_kelch(PVX_083080)      PIPKDLSESEALLKEAEFYGIKFLPFPLVFCMGGFDGVEYLNSMELLDISQQCWRMCTPM    462
PK_kelch(PKH_121080)      PIPKDLSESEALLKEAEFYGIKFLPFPLVFCMGGFDGVEYLNSMELLDISQQCWRMCTPM    462
                                                         #

PF_kelch(PF3D7_1343700)   STKKAYFGSAVLNNFLYVFGGNNYDYKALFETEVYDRLRDVWYVSSNLNIPRRNNCGVTS    536
PM2_PMkelch               STKKAYFGSAVLNNFLYVFGGNNYDYKALFETEVYDRLRDTWFVSSNLNIPRRNNCGVTS    516
PM1_PMkelch               STKKAYFGSAVLNNFLYVFGGNNYDYKALFETEVYDRLRDTWFVSSNLNIPRRNNCGVTS    499
PM1S_PMkelch              STKKAYFGSAVLNNFLYVFGGNNYDYKALFETEVYDRLRDTWFVSSNLNIPRRNNCGVTS    499
PM4_PMkelch               STKKAYFGSAVLNNFLYVFGGNNYDYKALFETEVYDRLRDTWFVSSNLNIPRRNNCGVTS    499
PM2848_PMkelch            STKKAYFGSAVLNNFLYVFGGNNYDYKALFETEVYDRLRDTWFVSSNLNIPRRNNCGVTS    499
PM454_PMkelch             STKKAYFGSAVLNNFLYVFGGNNYDYKALFETEVYDRLRDTWFVSSNLNIPRRNNCGVTS    499
PM5_PMkelch               STKKAYFGSAVLNNFLYVFGGNNYDYKALFETEVYDRLRDTWFVSSNLNIPRRNNCGVTS    499
PM1454_PMkelch            STKKAYFGSAVLNNFLYVFGGNNYDYKALFETEVYDRLRDTWFVSSNLNIPRRNNCGVTS    499
PMS_PMkelch               STKKAYFGSAVLNNFLYVFGGNNYDYKALFETEVYDRLRDTWFVSSNLNIPRRNNCGVTS    486
PM1381_PMkelch            STKKAYFGSAVLNNFLYVFGGNNYDYKALFETEVYDRLRDTWFVSSNLNIPRRNNCGVTS    486
PM17_PMkelch              STKKAYFGSAVLNNFLYVFGGNNYDYKALFETEVYDRLRDTWFVSSNLNIPRRNNCGVTS    486
```

```
PM18_PMkelch                  STKKAYFGSAVLNNFLYVFGGNNYDYKALFETEVYDRLRDTWFVSSNLNIPRRNNCGVTS     486
PM048_PMkelch                 STKKAYFGSAVLNNFLYVFGGNNYDYKALFETEVYDRLRDTWFVSSNLNIPRRNNCGVTS     486
PV_kelch(PVX_083080)          STKKAYFGSAVLNNFLYVFGGNNYDYKALFETEVYDRLRDTWFVSSNLNIPRRNNCGVTS     522
PK_kelch(PKH_121080)          STKKAYFGSAVLNNFLYVFGGNNYDYKALFETEVYDRLRDTWFVSSNLNIPRRNNCGVTS     522

PF_kelch(PF3D7_1343700)       NGRIYCIGGYDGSSIIPNVEAYDHRMKAWVEVAPLNTPRSSAMCVAFDNKIYVIGGTNGE     596
PM2_PMkelch                   NGRIYCIGGYDGSSIIPNVEAYDHRMKAWVEIAPLNTPRSSSMCVAFDNKIYVIGGTNGE     576
PM1_PMkelch                   NGRIYCIGGYDGSSIIPNVEAYDHRMKAWVEIAPLNTPRSSSMCVAFDNKIYVIGGTNGE     559
PM1S_PMkelch                  NGRIYCIGGYDGSSIIPNVEAYDHRMKAWVEIAPLNTPRSSSMCVAFDNKIYVIGGTNGE     559
PM4_PMkelch                   NGRIYCIGGYDGSSIIPNVEAYDHRMKAWVEIAPLNTPRSSSMCVAFDNKIYVIGGTNGE     559
PM2848_PMkelch                NGRIYCIGGYDGSSIIPNVEAYDHRMKAWVEIAPLNTPRSSSMCVAFDNKIYVIGGTNGE     559
PM454_PMkelch                 NGRIYCIGGYDGSSIIPNVEAYDHRMKAWVEIAPLNTPRSSSMCVAFDNKIYVIGGTNGE     559
PM5_PMkelch                   NGRIYCIGGYDGSSIIPNVEAYDHRMKAWVEIAPLNTPRSSSMCVAFDNKIYVIGGTNGE     559
PM1454_PMkelch                NGRIYCIGGYDGSSIIPNVEAYDHRMKAWVEIAPLNTPRSSSMCVAFDNKIYVIGGTNGE     559
PMS_PMkelch                   NGRIYCIGGYDGSSIIPNVEAYDHRMKAWVEIAPLNTPRSSSMCVAFDNKIYVIGGTNGE     546
PM1381_PMkelch                NGRIYCIGGYDGSSIIPNVEAYDHRMKAWVEIAPLNTPRSSSMCVAFDNKIYVIGGTNGE     546
PM17_PMkelch                  NGRIYCIGGYDGSSIIPNVEAYDHRMKAWVEIAPLNTPRSSSMCVAFDNKIYVIGGTNGE     546
PM18_PMkelch                  NGRIYCIGGYDGSSIIPNVEAYDHRMKAWVEIAPLNTPRSSSMCVAFDNKIYVIGGTNGE     546
PM048_PMkelch                 NGRIYCIGGYDGSSIILNVEAYDHRMKAWVEIAPLNTPRSSSMCVAFDNKIYVIGGTNGE     546
PV_kelch(PVX_083080)          NGRIYCIGGYDGSSIIPNVEAYDHRMKAWVEIAPLNTPRSSSMCVAFDNKIYVIGGTNGE     582
PK_kelch(PKH_121080)          NGRIYCIGGYDGSCIIPNVEAYDHRMKAWVEIAPLNTPRSSSMCVAFENKIYVIGGTNGE     582
                                #             *                          #

PF_kelch(PF3D7_1343700)       RLNSIEVYEEKMNKWEQFPYALLEARSSGAAFNYLNQIYVVGGIDNEHNILDSVEQYQPF     656
PM2_PMkelch                   RLNSIEVYEEKMNKWEQFPYALLEARSSGAAFNYLNQIYVVGGIDNEHNILDSVEQYQPF     636
PM1_PMkelch                   RLNSIEVYEEKMNKWEQFPYALLEARSSGAAFNYLNQIYVVGGIDNEHNILDSVEQYQPF     619
PM1S_PMkelch                  RLNSIEVYEEKMNKWEQFPYALLEARSSGAAFNYLNQIYVVGGIDNEHNILDSVEQYQPF     619
PM4_PMkelch                   RLNSIEVYEEKMNKWEQFPYALLEARSSGAAFNYLNQIYVVGGIDNEHNILDSVEQYQPF     619
PM2848_PMkelch                RLNSIEVYEEKMNKWEQFPYALLEARSSGAAFNYLNQIYVVGGIDNEHNILDSVEQYQPF     619
PM454_PMkelch                 RLNSIEVYEEKMNKWEQFPYALLEARSSGAAFNYLNQIYVVGGIDNEHNILDSVEQYQPF     619
PM5_PMkelch                   RLNSIEVYEEKMNKWEQFPYALLEARSSGAAFNYLNQIYVVGGIDNEHNILDSVEQYQPF     619
PM1454_PMkelch                RLNSIEVYEEKMNKWEQFPYALLEARSSGAAFNYLNQIYVVGGIDNEHNILDSVEQYQPF     619
PMS_PMkelch                   RLNSIEVYEEKMNKWEQFPYALLEARSSGAAFNYLNQIYVVGGIDNEHNILDSVEQYQPF     606
PM1381_PMkelch                RLNSIEVYEEKMNKWEQFPYALLEARSSGAAFNYLNQIYVVGGIDNEHNILDSVEQYQPF     606
PM17_PMkelch                  RLNSIEVYEEKMNKWEQFPYALLEARSSGAAFNYLNQIYVVGGIDNEHNILDSVEQYQPF     606
PM18_PMkelch                  RLNSIEVYEEKMNKWEQFPYALLEARSSGAAFNYLNQIYVVGGIDNEHNILDSVEQYQPF     606
PM048_PMkelch                 RLNSIEVYEEKMNKWEQFPYALLEARSSGAAFNYLNQIYVVGGIDNEHNILDSVEQYQPF     606
PV_kelch(PVX_083080)          RLNSIEVYDEKMNKWEQFPYALLEARSSGAAFNYLNQIYVVGGIDNEHNILDSVEQYQPF     642
PK_kelch(PKH_121080)          RLNSIEVYDEKMNKWEQFPYALLEARSSGAAFNYLNQIYVVGGIDNEHNILDSVEQYQPF     642

PF_kelch(PF3D7_1343700)       NKRWQFLNGVPEKKMNFGAATLSDSYIITGGENGEVLNSCHFFSPDTNEWQLGPSLLVPR     716
PM2_PMkelch                   NKRWQFLNGVPEKKMNFGASTLSDSYIITGGENGEVLNSCHFFSPDTNEWQIGPSLLVP-     695
PM1_PMkelch                   NKRWQFLNGVPEKKMNFGASTLSDSYIITGGENGEVLNSCHFFSPDTNEWQIGPSLLVP-     678
PM1S_PMkelch                  NKRWQFLNGVPEKKMNFGASTLSDSYIITGGENGEVLNSCHFFSPDTNEWQIGPSLLVP-     678
PM4_PMkelch                   NKRWQFLNGVPEKKMNFGASTLSDSYIITGGENGEVLNSCHFFSPDTNEWQIGPSLLVP-     678
PM2848_PMkelch                NKRWQFLNGVPEKKMNFGASTLSDSYIITGGENGEVLNSCHFFSPDTNEWQIGPSLLVP-     678
PM454_PMkelch                 NKRWQFLNGVPEKKMNFGASTLSDSYIITGGENGEVLNSCHFFSPDTNEWQIGPSLLVP-     678
PM5_PMkelch                   NKRWQFLNGVPEKKMNFGASTLSDSYIITGGENGEVLNSCHFFSPDTNEWQIGPSLLVP-     678
PM1454_PMkelch                NKRWQFLNGVPEKKMNFGASTLSDSYIITGGENGEVLNSCHFFSPDTNEWQIGPSLLVP-     678
PMS_PMkelch                   NKRWQFLNGVPEKKMNFGASTLSDSYIITGGENGEVLNSCHFFSPDTNEWQIGPSLLVP-     665
PM1381_PMkelch                NKRWQFLNGVPEKKMNFGASTLSDSYIITGGENGEVLNSCHFFSPDTNEWQIGPSLLVP-     665
PM17_PMkelch                  NKRWQFLNGVPEKKMNFGASTLSDSYIITGGENGEVLNSCHFFSPDTNEWQIGPSLLVP-     665
PM18_PMkelch                  NKRWQFLNGVPEKKMNFGASTLSDSYIITGGENGEVLNSCHFFSPDTNEWQIGPSLLVP-     665
PM048_PMkelch                 NKRWQFLNGVPEKKMNFGASTLSDSYIITGGENGEVLNSCHFFSPDTNEWQIGPS-----     661
PV_kelch(PVX_083080)          NKRWQFLNGVPEKKMNFGAATLSDSYIITGGENGDVLNSCHFFSPDTNEWQIGPSLLVPR     702
PK_kelch(PKH_121080)          NKRWQFLNGVPEKKMNFGAATLSDSYIITGGENGDVLNSCHFFSPDTNEWQIGPSLLVPR     702

PF_kelch(PF3D7_1343700)       FGHSVLIANI       726
PM2_PMkelch                   ----------       695
PM1_PMkelch                   ----------       678
PM1S_PMkelch                  ----------       678
PM4_PMkelch                   ----------       678
PM2848_PMkelch                ----------       678
PM454_PMkelch                 ----------       678
PM5_PMkelch                   ----------       678
PM1454_PMkelch                ----------       678
PMS_PMkelch                   ----------       665
PM1381_PMkelch                ----------       665
PM17_PMkelch                  ----------       665
PM18_PMkelch                  ----------       665
PM048_PMkelch                 ----------       661
PV_kelch(PVX_083080)          FGHSVLIANI       712
PK_kelch(PKH_121080)          FGHSVLIANI       712
```

Figure S1. Alignment of amino acid of *P. malariae* and the other *Plasmodium spp. kelch* propeller gene. Mutation found in *P. malariae* isolate indicated by an asterisk (*) and dark grey shading. Light grey shading and # was used to indicate common mutation of *P. falciparum* (F446I, R539T and C580Y).

```
PF_kelch(PF3D7_1343700)       MEGEKVKTKANSISNFSMTYDRESGGNSNSDDKSG-SSSENDSNSFMNLTSDKNEKTENN     59
POw1_POkelch                  -----------------------------SDEKSMSSSDENESSSFMNLTSDKNEKIENN     31
POc13_POkelch                 ---------------------------------------------------------ENN     3
POw20_POkelch                 ---------------------------------------------------------ENN     3
POw21_POkelch                 -----------------------------------------------------------N     1
POw23_POkelch                 -----------------------------------------------------------N     1
PV_kelch(PVX_083080)          MEGEKI--KSNSISNFSVTYERESGANSNSDDKS-VSSSENESNSFMNLTSDKNEKTENN     57
PK_kelch(PKH_121080)          MEDEKI--KSNSISNFSVTYERESGANSNSDDKS-VSSSENESNSFMNLTSDKNEKTENN     57

PF_kelch(PF3D7_1343700)       SFLLNNSSYGNVKDSLLESIDMSVLDSNFDSKKDFLPSNLSRTFNNMSKDNIGNKYLNKL     119
POw1_POkelch                  SFLLNNSSFANMKDSLLESIDLSVLDSNFDSKKDFLPSNFSKNFNNLSKENISNKYLNKL     91
POc13_POkelch                 SFLLNNSSFANMKDSLLESIDLSVLDSNFDSKKDFLPSNFSKNFNNLSKENISNKYLNKL     63
POw20_POkelch                 SFLLNNSSFANMKDSLLESIDLSVLDSNFDSKKDFLPSNFSKNFNNLSKENISNKYLNKL     63
POw21_POkelch                 SFLLNNSSFANMKDSLLESIDLSVLDSNFDSKKDFLPSNFSKNFNNLSKENISNKYLNKL     61
POw23_POkelch                 SFLLNNSSFANMKDSLLESIDLSVLDSNFDSKKDFLPSNFSKNFNNLSKENISNKYLNKL     61
PV_kelch(PVX_083080)          SFILNNSSFANMKDSLLESIDLSVLDSNFDSKKDFLPSNLSKNFNNLSKENLGNKYLNKL     117
PK_kelch(PKH_121080)          SFILNNSSFANMKDSFLESIDLSILDSNFDSKKDFLPSNLSKNFNNLSKENLGNKYLNKL     117

PF_kelch(PF3D7_1343700)       LNKKKDTITNENNNINHNNNNNNLTANNITNNLINNNMNSPSIMNTNKKENFLDAANLIN     179
POw1_POkelch                  LNKSDSLFMSKNKDLNLTDVSNNLNNSNSNS---HGNGTNVPLRNNNNRKDSFMDSQNPLN     148
POc13_POkelch                 LNKSDSLFMSKNKDINLTDVSNNLNNSNSNS---HGNGANVPMRNNNNRKDSFMDSQNPLN     120
POw20_POkelch                 LNKSDSLFMSKNKDLNLTDVSNNLNNSNSNS---HGNGTNVPLRNNNNRRDSFMDSQNPLN     120
POw21_POkelch                 LNKSDSLFMSKNKDLNLTDVSNNLNNSNSNS---HGNGTNVPLRNNNNRKDSFMDSQNPLN     118
POw23_POkelch                 LNKSDSLFMSKNKDLNLTDVSNNLNNSNSNS---HGNGTNVPLRNNNNRKDSFMDSQNPLN     118
PV_kelch(PVX_083080)          LNKSDSMFMSKGKDMNLMENNL--------------GSNNLPVKSSNKKEGFMDSSTPIN     163
PK_kelch(PKH_121080)          LNKSDSLFMSKNKDMNLMDNNM--------------GSNNLPVKSSNRKEGFMDSSTPIN     163
                                                                               *

PF_kelch(PF3D7_1343700)       ---DDSGLNNLKKFSTVNNVNDTYEKKIIETELSDASDFENMVGDLRITFINWLKKTQMN     236
POw1_POkelch                  TNEENNALNNLKKYTNANNINDTYEKKIIETELSDSSDFENMVGDLRITFINWLKKTQMN     208
POc13_POkelch                 VNEENNALNNLKKYANTNNINDTYEKKIIETELSDSSDFENMVGDLRITFINWLKKTQMN     180
POw20_POkelch                 TNEENNALNNLKKYTNANNINDTYEKKIIETELSDSSDFENMVGDLRITFINWLKKTQMN     180
POw21_POkelch                 TNEENNALNNLKKYTNANNINDTYEKKIIETELSDSSDFENMVGDLRITFINWLKKTQMN     178
POw23_POkelch                 TNEENNALNNLKKYTNANNINDTYEKKIIETELSDSSDFENMVGDLRITFINWLKKTQMN     178
PV_kelch(PVX_083080)          ANE-DNAMNNLKKYSNANNINDTYEKKIIETELSDSSDFENMVGDLRITFINWLKKTQMN     222
PK_kelch(PKH_121080)          ANE-DNAMNNLKKYSNTNNINDTYEKKIIETELSDSSDFENMVGDLRITFINWLKKTQMN     222

PF_kelch(PF3D7_1343700)       FIREKDKLFKDKKELEMERVRLYKELENRKNIEEQKLHDERKKLDIDISNGYKQIKKEKE     296
POw1_POkelch                  FIREKDKLFKDKKELEMERIRLYKEIENRKAIEEQKLYDERKKLDIDISNGYKQIKKEKE     268
POc13_POkelch                 FIREKDKLFKDKKELEMERIRLYKEIENRKAIEEQKLYDERKKLDIDISNGYKQIKKEKE     240
POw20_POkelch                 FIREKDKLFKDKKELEMERIRLYKEIENRKAIEEQKLYDERKKLDIDISNGYKQIKKEKE     240
POw21_POkelch                 FIREKDKLFKDKKELEMERIRLYKEIENRKAIEEQKLYDERKKLDIDISNGYKQIKKEKE     238
POw23_POkelch                 FIREKDKLFKDKKELEMERIRLYKEIENRKAIEEQKLYDERKKLDIDISNGYKQIKKEKE     238
PV_kelch(PVX_083080)          FIREKDKLFKDKKELEMERIRLYKEIENRKSIEEQKLHDERKKLDIDISNGYKQIKKEKE     282
PK_kelch(PKH_121080)          FIREKDKLFKDKKELEMERIRLYKEIENRKSIEEQKLHDERKKLDIDISNGYKQIKKEKE     282

PF_kelch(PF3D7_1343700)       EHRKRFDEERLRFLQEIDKIKLVLYLEKEKYYQEYKNFENDKKKIVDANIATETMIDINV     356
POw1_POkelch                  EHRKRFDEERLRFLQEIDKIKLVLYLEKEKYFQEYKNFENDKKKIVDANIATETMIDINV     328
POc13_POkelch                 EHRKRFDEERLRFLQEIDKIKLVLYLEKEKYFQEYKNFENDKKKIVDANIATETMIDINV     300
POw20_POkelch                 EHRKRFDEERLRFLQEIDKIKLVLYLEKEKYFQEYKNFENDKKKIVDANIATETMIDINV     300
POw21_POkelch                 EHRKRFDEERLRFLQEIDKIKLVLYLEKEKYFQEYKNFENDKKKIVDANIATETMIDINV     298
POw23_POkelch                 EHRKRFDEERLRFLQEIDKIKLVLYLEKEKYFQEYKNFENDKKKIVDANIATETMIDINV     298
PV_kelch(PVX_083080)          EHRKRFDEERLRFLQEIDKIKLVLYLEKEKYFQEYKNFENDKKKIVDANIATETMIDINV     342
PK_kelch(PKH_121080)          EHRKRFDEERLRFLQEIDKIKLVLYLEKEKYFQEYKNFENDKKKIVDANIATETMIDINV     342

PF_kelch(PF3D7_1343700)       GGAIFETSRHTLTQQKDSFIEKLLSGRHHVTRDKQGRIFLDRDSELFRIILNFLRNPLTI     416
POw1_POkelch                  GGAIFETSRHTLTQQKDSFIEKLLSGRYHVTRDKQGRIFLDRDSELFRIILNFLRNPLTV     388
POc13_POkelch                 GGAIFETSRHTLTQQKDSFIEKLLSGRYHVTRDKQGRIFLDRDSELFRIILNFLRNPLTV     360
POw20_POkelch                 GGAIFETSRHTLTQQKDSFIEKLLSGRYHVTRDKQGRIFLDRDSELFRIILNFLRNPLTV     360
POw21_POkelch                 GGAIFETSRHTLTQQKDSFIEKLLSGRYHVTRDKQGRIFLDRDSELFRIILNFLRNPLTV     358
POw23_POkelch                 GGAIFETSRHTLTQQKDSFIEKLLSGRYHVTRDKQGRIFLDRDSELFRIILNFLRNPLTV     358
```

```
PV_kelch(PVX_083080)        GGAIFETSRHTLTQQKDSFIEKLLSGRYHVTRDKQGRIFLDRDSELFRIILNFLRNPLTV     402
PK_kelch(PKH_121080)        GGAIFETSRHTLTQQKDSFIEKLLSGRYHVTRDKQGRIFLDRDSELFRIILNFLRNPLTV     402

PF_kelch(PF3D7_1343700)     PIPKDLSESEALLKEAEFYGIKFLPFPLVFCIGGFDGVEYLNSMELLDISQQCWRMCTPM     476
POw1_POkelch                PIPKDLSESEALLKEAEFYGIKFLPFPLVFCIGGFDGVEYLNSMELLDISQQCWRMCTPM     448
POc13_POkelch               PIPKDLSESEALLKEAEFYGIKFLPFPLVFCIGGFDGVEYLNSMELLDISQQCWRMCTPM     420
POw20_POkelch               PIPKDLSESEALLKEAEFYGIKFLPFPLVFCIGGFDGVEYLNSMELLDISQQCWRMCTPM     420
POw21_POkelch               PIPKDLSESEALLKEAEFYGIKFLPFPLVFCIGGFDGVEYLNSMELLDISQQCWRMCTPM     418
POw23_POkelch               PIPKDLSESEALLKEAEFYGIKFLPFPLVFCIGGFDGVEYLNSMELLDISQQCWRMCTPM     418
PV_kelch(PVX_083080)        PIPKDLSESEALLKEAEFYGIKFLPFPLVFCMGGFDGVEYLNSMELLDISQQCWRMCTPM     462
PK_kelch(PKH_121080)        PIPKDLSESEALLKEAEFYGIKFLPFPLVFCMGGFDGVEYLNSMELLDISQQCWRMCTPM     462
                                                         #

PF_kelch(PF3D7_1343700)     STKKAYFGSAVLNNFLYVFGGNNYDYKALFETEVYDRLRDVWYVSSNLNIPRRNNCGVTS     536
POw1_POkelch                STKKAYFGSAVLNNFLYVFGGNNYDYKALFETEVYDRLRDTWFVSSNLNIPRRNNCGVTS     508
POc13_POkelch               TTKKAYFGSAVLNNFLYVFGGNNYDYKALFETEVYDRLRDTWFVSSNLNIPRRNNCGVTS     480
POw20_POkelch               STKKAYFGSAVLNNFLYVFGGNNYDYKALFETEVYDRLRDTWFVSSNLNIPRRNNCGVTS     480
POw21_POkelch               STKKAYFGSAVLNNFLYVFGGNNYDYKALFETEVYDRLRDTWFVSSNLNIPRRNNCGVTS     478
POw23_POkelch               STKKAYFGSAVLNNFLYVFGGNNYDYKALFETEVYDRLRDTWFVSSNLNIPRRNNCGVTS     478
PV_kelch(PVX_083080)        STKKAYFGSAVLNNFLYVFGGNNYDYKALFETEVYDRLRDTWFVSSNLNIPRRNNCGVTS     522
PK_kelch(PKH_121080)        STKKAYFGSAVLNNFLYVFGGNNYDYKALFETEVYDRLRDTWFVSSNLNIPRRNNCGVTS     522

PF_kelch(PF3D7_1343700)     NGRIYCIGGYDGSSIIPNVEAYDHRMKAWVEVAPLNTPRSSAMCVAFDNKIYVIGGTNGE     596
POw1_POkelch                NGRIYCIGGYDGSSIIPNVEAYDHRMKAWVEVAPLNTPRSSSMCVAFDNKIYVIGGTNGE     568
POc13_POkelch               NGRIYCIGGYDGSSIIPNVEAYDHRMKAWVEVAPLNTPRSSSMCVAFDNKIYVIGGTNGE     540
POw20_POkelch               NGRIYCIGGYDGSSIIPNVEAYDHRMKAWVEVAPLNTPRSSSMCVAFDNKIYVIGGTNGE     540
POw21_POkelch               NGRIYCIGGYDGSSIIPNVEAYDHRMKAWVEVAPLNTPRSSSMCVAFDNKIYVIGGTNGE     538
POw23_POkelch               NGRIYCIGGYDGSSIIPNVEAYDHRMKAWVEVAPLNTPRSSSMCVAFDNKIYVIGGTNGE     538
PV_kelch(PVX_083080)        NGRIYCIGGYDGSSIIPNVEAYDHRMKAWVEIAPLNTPRSSSMCVAFDNKIYVIGGTNGE     582
PK_kelch(PKH_121080)        NGRIYCIGGYDGSCIIPNVEAYDHRMKAWVEIAPLNTPRSSSMCVAFENKIYVIGGTNGE     582
                              #                                         #

PF_kelch(PF3D7_1343700)     RLNSIEVYEEKMNKWEQFPYALLEARSSGAAFNYLNQIYVVGGIDNEHNILDSVEQYQPF     656
POw1_POkelch                RLNSIEVYDEKMNKWEQFPYALLEARSSGAAFNYLNQIYVVGGIDNEHNILDSVEQYQPF     628
POc13_POkelch               RLNSIEVYDEKMNKWEQFPYALLEARSSGAAFNYLNQIYVVGGIDNEHNILDSVEQYQPF     600
POw20_POkelch               RLNSIEVYDEKMNKWEQFPYALLEARSSGAAFNYLNQIYVVGGIDNEHNILDSVEQYQPF     600
POw21_POkelch               RLNSIEVYDEKMNKWEQFPYALLEARSSGAAFNYLNQIYVVGGIDNEHNILDSVEQYQPF     598
POw23_POkelch               RLNSIEVYDEKMNKWEQFPYALLEARSSGAAFNYLNQIYVVGGIDNEHNILDSVEQYQPF     598
PV_kelch(PVX_083080)        RLNSIEVYDEKMNKWEQFPYALLEARSSGAAFNYLNQIYVVGGIDNEHNILDSVEQYQPF     642
PK_kelch(PKH_121080)        RLNSIEVYDEKMNKWEQFPYALLEARSSGAAFNYLNQIYVVGGIDNEHNILDSVEQYQPF     642

PF_kelch(PF3D7_1343700)     NKRWQFLNGVPEKKMNFGAATLSDSYIITGGENGEVLNSCHFFSPDTNEWQLGPSLLVPR     716
POw1_POkelch                NKRWQFLNGVPEKKMNFGSATLSDSYIITGGENGDVLNSCHFFSPDTNEWQIGPSLLVP-     687
POc13_POkelch               NKRWQFLNGVPEKKMNFGSATLSDSYIITGGENGDVLNSCHFFSPDTNEWQIGPSLLVP-     659
POw20_POkelch               NKRWQFLNGVPEKKMNFGSATLSDSYIITGGENGDVLNSCHFFSPDTNEWQIGPSLLVP-     659
POw21_POkelch               NKRWQFLNGVPEKKMNFGSATLSDSYIITGGENGDVLNSCHFFSPDTNEWQIGPSLLVP-     657
POw23_POkelch               NKRWQFLNGVPEKKMNFGSATLSDSYIITGGENGDVLNSCHFFSPDTNEWQIGPSLLVP-     657
PV_kelch(PVX_083080)        NKRWQFLNGVPEKKMNFGAATLSDSYIITGGENGDVLNSCHFFSPDTNEWQIGPSLLVPR     702
PK_kelch(PKH_121080)        NKRWQFLNGVPEKKMNFGAATLSDSYIITGGENGDVLNSCHFFSPDTNEWQIGPSLLVPR     702

PF_kelch(PF3D7_1343700)     FGHSVLIANI       726
POw1_POkelch                ----------       687
POc13_POkelch               ----------       659
POw20_POkelch               ----------       659
POw21_POkelch               ----------       657
POw23_POkelch               ----------       657
PV_kelch(PVX_083080)        FGHSVLIANI       712
PK_kelch(PKH_121080)        FGHSVLIANI       712
```

Figure S2. Alignment of amino acid of *P. ovale* and the other *Plasmodium spp. kelch* propeller gene. Mutation found in *P. ovale* isolate indicated by an asterisk (*) and dark grey shading. Light grey shading and # was used to indicate common mutation of *P. falciparum* (F446I, R539T and C580Y).
